# Adult-Age Inflammatory Pain Experience Enhances Long-Term Pain Vigilance in Rats

**DOI:** 10.1371/journal.pone.0036767

**Published:** 2012-05-04

**Authors:** Sheng-Guang Li, Jin-Yan Wang, Fei Luo

**Affiliations:** 1 Key Laboratory of Mental Health, Institute of Psychology, Chinese Academy of Sciences, Beijing, China; 2 The Graduate University, Chinese Academy of Sciences, Beijing, China; University of Bologna, Italy

## Abstract

**Background:**

Previous animal studies have illustrated a modulatory effect of neonatal pain experience on subsequent pain-related behaviors. However, the relationship between chronic pain status in adulthood and future pain perception remains unclear.

**Methodology/Principal Findings:**

In the current study, we investigated the effects of inflammatory pain experience on subsequent formalin-evoked pain behaviors and fear conditioning induced by noxious stimulation in adult rats. Our results demonstrated an increase of the second but not the first phase of formalin-induced pain behaviors in animals with a history of inflammatory pain that have recovered. Similarly, rats with persistent pain experience displayed facilitated acquisition and prolonged retention of pain-related conditioning. These effects of prior pain experience on subsequent behavior were prevented by repeated morphine administration at an early stage of inflammatory pain.

**Conclusions/Significance:**

These results suggest that chronic pain diseases, if not properly and promptly treated, may have a long-lasting impact on processing and perception of environmental threats. This may increase the susceptibility of patients to subsequent pain-related disorders, even when chronic pain develops in adulthood. These data highlight the importance of treatment of chronic pain at an early stage.

## Introduction

It is a long-held belief that pain is a highly individual and subjective experience [1]. Much of this variation may result from the integration of past experiences and future predictions about noxious stimulation [2]. The experience of pain significantly affects the physiological, as well as psychological states of individuals, resulting in anxiety, depression, and even cognitive deficits [3], [4], [5]. A sizable body of clinical literature indicates that people with a history of persistent pain exhibit increased responsiveness to noxious events, evidenced by changes in pain threshold [6], [7], pain ratings [8], [9], and emotional and cognitive responses [10]. Moreover, once beliefs and expectancies about pain are formed, they become stable and difficult to modify, even after the injury has resolved [11].

Converging evidence from animal studies supports a role of previous experience in pain perception [12], [13], [14], [15]. Early pain experiences result in long-lasting and potentially detrimental alterations in nociceptive systems. Both long-term hypoalgesia and hyperalgesia have been observed as a result of neonatal noxious experience, suggesting global abnormalities of pain regulatory mechanisms [12], [14], [16], [17]. Ruda et al. [15] investigated long-term effects of early pain in rats. They found that rats that had received an intraplantar hind paw injection of complete Freund's adjuvant (CFA) in the post-neonatal period showed a significantly enhanced nocifensive response in the formalin test and exacerbated thermal hyperalgesia when re-inflamed by new CFA injection as adults. These findings were corroborated by another study, which showed that long-term excessive thermal or mechanical hyperalgesia after inflammation manifested not only in the hind paw that received carrageenan in the neonatal period, but also in the uninjured paw [14]. With conditioning paradigms, some studies also assessed the effects of prior pain experiences on pain-related emotional responses [18], [19], [20]. For example, Hummel et al. found that young rats (less than 150 g) subjected to neuropathic injury or inflammatory insult displayed a significant increase in conditioned place aversion to a pain-paired environment; this response was prevented by systemic morphine treatment prior to conditioning trials [18]. Using adult male and female rats in separate studies, Aliosi et al. also demonstrated long-term consequences of adult exposure to formalin pain by investigating the effects of gonadectomy and repeated formalin treatment on behavioral responses. They found that the effects were sex-dependent and modulated by gonadal hormones in both males and females [21], [22], [23].

Despite the many animal studies of the modulatory effects of pain experience on subsequent pain behaviors, at least two important issues remain unresolved. First, most available evidence derives from studies of the effects of neonatal injury on pain responses. The ability of chronic pain experience in adulthood to shape later pain perception and responses is largely unknown. Second, very few studies have examined the relationship between pain experience and later pain expectancy. To address these issues, the present study investigated the effects of an inflammatory pain history on subsequent formalin-evoked pain behaviors, as well as tone-elicited anticipatory pain responses, in adult rats. A trace conditioning paradigm was applied to associate an auditory cue with a noxious laser stimulus, and correlation analysis was used to examine the relationship between pain sensitivity and later anticipatory responses.

## Materials and Methods

### Animals

Eighty-two male Sprague-Dawley rats (180–200 g, 8 weeks of age) from the Laboratory Animal Center of the Academy of Military Medical Sciences were used in this study. All rats were housed individually and maintained on a reverse 12 h light cycle (lights on at 7:00 P.M.). Food and water were available *ad libitum*. Rats were handled twice a day and were acclimatized to the experimental apparatus for 1 h before each behavioral procedure. Experimental protocols were approved by the Institutional Review Board of the Institute of Psychology, Chinese Academy of Sciences (confirmation number: A09013) and were in strict accordance with the National Institutes of Health Guide for Care and Use of Laboratory Animals. All attempts were made to minimize the number of animals used and to avoid any undue suffering.

### Drugs

Complete Freund's adjuvant (CFA; Sigma, St. Louis, MO) was delivered in a volume of 100 µl per rat. Morphine hydrochloride (Qinghai Pharmaceutical Factory, Qinghai, China) was dissolved in sterile saline (0.9% NaCl) at a concentration of 10 mg/ml and was administered at 5 mg/kg, s.c. to reduce pain associated with chronic inflammation [24], [25]. Stock formaldehyde solution (37% formaldehyde or 100% formalin; Beijing Chemical Reagents Company, Beijing, China) was diluted to 5% (v/v) formalin in isotonic saline.

### Experimental design

Two experiments were conducted in this study. We used a CFA-induced inflammatory pain model to investigate the effects of chronic pain history on subsequent pain behavior (experiment 1) and pain expectancy (experiment 2).

**Figure 1:**
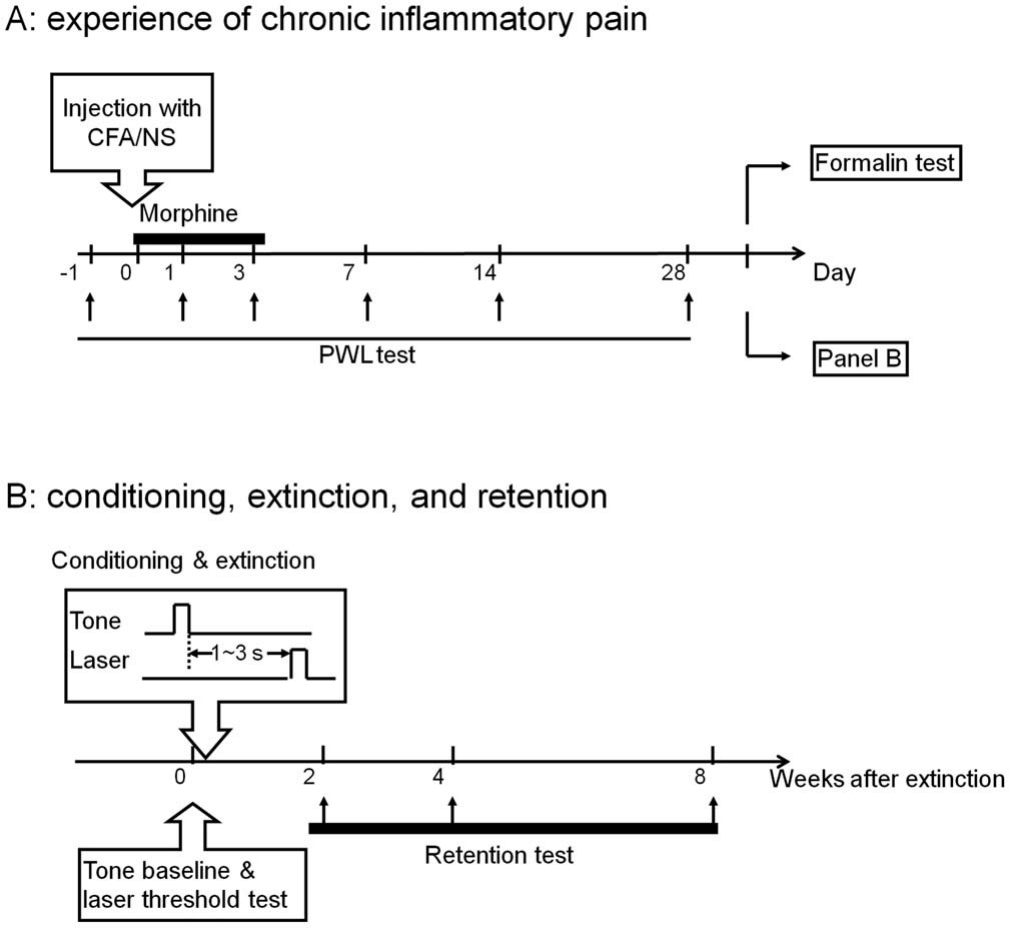
Schematic representation of the experimental design. (A) Chronic inflammatory pain experience. Animals received an intraplantar injection of complete Freund's adjuvant (CFA) or normal saline (NS) on day 0. A subset of CFA animals were given morphine (5 mg/kg, s.c.) twice a day for 4 consecutive days, as indicated by the thick horizontal bar. Paw withdrawal latency (PWL) tests were performed 1 day before, and 1, 3, 7, 14, and 28 days after injection, as indicated by arrows. On day 31 after CFA inoculation, a formalin test was conducted with 5% formalin solution (s.c.) into the intact hind paw to produce spontaneous pain (experiment 1). (B) Conditioning, extinction and retention (experiment 2). A tone-laser conditioning paradigm with 1–3 s variable intervals was applied for 40 trials, followed by an immediate extinction test of 30 trials and long-term retention tests at weeks 2, 4, and 8 after extinction, as indicated by arrows.

Rats were divided into three groups: normal saline (NS, n = 18), CFA (n = 48), and CFA plus morphine (CFA+MOR, n = 16), according to the initial treatment on day 0 (i.e., intraplantar injection of saline for the NS group and CFA for the other two groups). Rats in the CFA+MOR group received morphine, s.c. twice per day (12-h interval) for 4 consecutive days (day 0, first injection at 1 h before CFA inoculation; day 1–3, first injection at 6 h before pain test). Animals were then allowed to recover for 30 days and underwent a formalin test (experiment 1) or pain-related fear conditioning (experiment 2) on day 31 (Fig. 1A). The design was between groups, such that each rat participated in only one experiment and one treatment group.

In the formalin test, all groups of rats (NS, CFA, and CFA+MOR, n = 8/group) received 5% formalin, s.c. into the intact hind paws. During the conditioning procedure, tone-laser pairings and extinction took place in consecutive sessions. Baseline orientating reactions (ORs) to auditory stimuli and nociceptive thresholds of laser pulses were measured before conditioning. The rats in the CFA group were further divided into CFA/conditioning (n = 26) and CFA/random (n = 14) subgroups, receiving tone-laser pairing and randomly sequenced tone and laser stimulations, respectively. In the CFA/conditioning group, the hind paws ipsilateral (CFA/ip, n = 17) or contralateral (CFA/con, n = 9) to the previous CFA injection were tested separately. In the conditioning session, 40 tone-laser pairings were presented, followed by an immediate 30-trial extinction test to evaluate the acquisition of a conditioned response (i.e., anticipatory pain behavior). Retention tests were performed at 2, 4, and 8 weeks after the extinction session to assess maintenance of the negative affect (Fig. 1B).

### Pain tests

#### Chronic inflammatory pain

Chronic inflammation and hyperalgesia were elicited by intraplantar injection of CFA into the rat hindpaw [26]. Control rats received the same volume of saline. The CFA/NS injection was counterbalanced across left and right hind paws. Paw withdrawal latency (PWL) was measured 1 day before (baseline), and 1, 3, 7, 14, and 28 days after CFA or NS administration. A beam of light from a radiant heat apparatus (100-W projector lamp) was focused on the plantar surface of the hindpaw, and PWL was defined as the time between light onset and paw lift. The intensity of light was adjusted to achieve PWLs around 8 s at baseline. A cut-off time of 22 s was used to avoid tissue damage. For each rat, the PWL was tested five times at 5-min intervals. Latency was calculated as the mean of four trials, excluding the first familiarization trial.

#### Formalin test

Rats received 5% formalin (50 µl, s.c.) into the plantar surface of the intact hind paw. Nociceptive behaviors were videotaped for 60 min and quantified by measuring the time spent biting or licking the injected paw in each 5-min intervals.

### Tone-laser conditioning

#### Apparatus and stimuli

Rats were placed in a custom-designed Plexiglas chamber (22×22×30 cm) with holes (3 mm diameter, 3 mm intervals) in the bottom. Tones (80 dB, 2900 Hz, 100-ms duration) from a speaker on the back wall of the chamber were used as conditioned stimuli (CS). A surgical CO_2_ laser stimulator (Model DM-300, Changchun Institute of Optics, Fine Mechanics and Physics, Chinese Academy of Science) was employed to generate the unconditioned stimulus (US), a noxious laser radiation beam in the infrared spectrum (10.6-µm wave length, 20 ms pulse width). The laser beam was applied to the plantar surface of the rat hind paw with a 1-mm diameter. To avoid habitation, sensitization, or skin damage, the location of the stimulation site was varied. The chamber was cleaned with 75% alcohol between rats.

The laser power was set to the withdrawal threshold to ensure an equivalent sensation level for each rat. A ramping procedure was used to measure the threshold intensity of laser pulses, as described by Brown et al. [27]. Briefly, rats were presented with laser stimuli beginning at 0.5 W, which was increased by 0.5 W until significant withdrawal behavior was observed.

#### Behavioral assessment

The laser- or tone-elicited behavior was scored according to Fan et al., who proposed five components of nocifensive behaviors: head turning, flinching, withdrawal, licking, and body movement [28], [29]. Head turning included shaking or elevating the head. Flinching involved a small abrupt jerking body movement (≤1 cm). Withdrawal was recorded when a rat retracted its paw by ≥1 cm away from the stimulus. Licking was scored when a rat retracted the paw ≥1 cm and licked it. Body movement involved body turning and running. In the present study, the behavioral scoring criteria were modified as follows: 0, immobility; 1, head turning or ear pricking; 2, flinching; 3, withdrawal, licking, and body movement. Behavior was videotaped, only the maximum score was recorded within each trial, and behavioral responses were assessed by cumulative scores every 5 successive trials.

#### Conditioning

A modified trace conditioning paradigm was applied to associate the auditory cue with the laser stimulus (Fig. 1B). In each pairing trial, the tone cue was presented 1–3 s before laser stimulation. The average inter-trial interval was 75 s (range 60–90 s).

### Statistical analysis

Data were expressed as means ± SEM. GraphPad *Prism* 5.01 (GraphPad Software, Inc., La Jolla, CA) and *Statistica* 6.0 were used for statistical analyses and graph generation. Data affected by two or three factors were analyzed with multifactor analysis of variance (ANOVA). *Student-Newman-Keuls* tests were used for post hoc comparisons. The relationship between pain threshold and later anticipatory response was examined using *Pearson* correlations. Results were considered statistically significant if *P*<0.05.

## Results

### Chronic pain experience facilitates later pain behaviors in the formalin test

**Figure 2:**
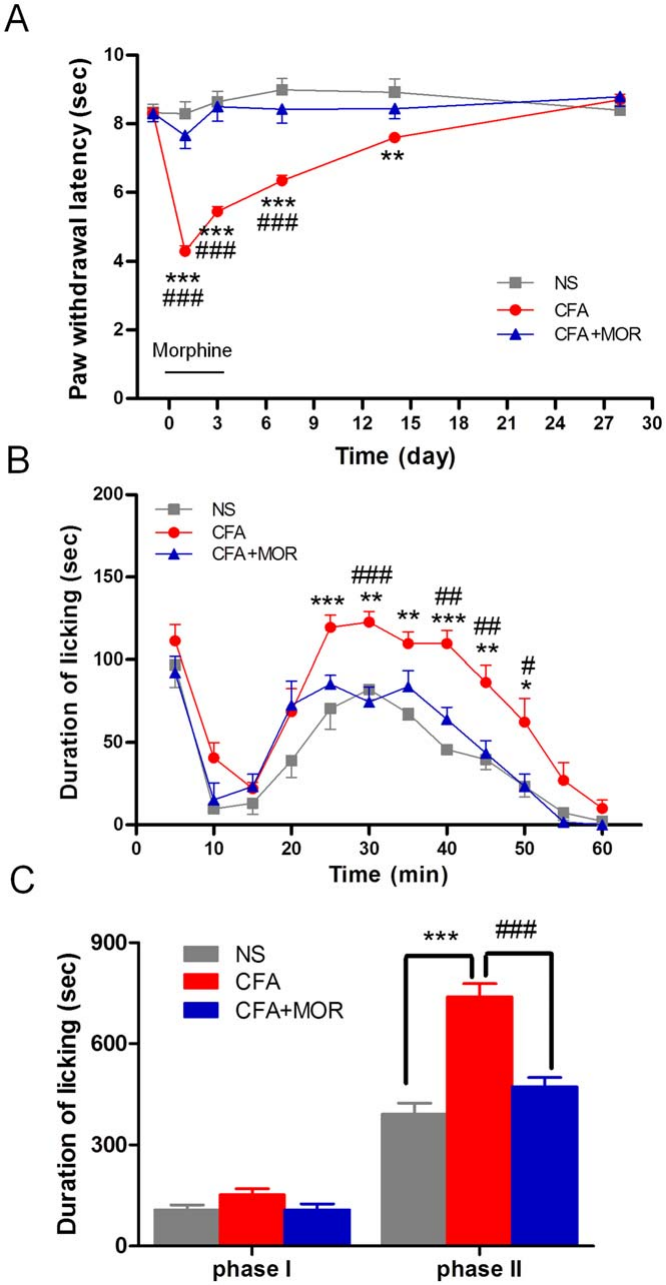
Effect of chronic pain history on formalin pain. (A) The time course of CFA-induced thermal hyperalgesia. A significant decrease was observed in PWL of the CFA-injected paw starting at day 1 and persisting at least 14 days, compared with baseline and saline control. Morphine treatment over day 0–3 prevented CFA-induced hyperalgesia. (B) Time-effect curve of chronic pain experience on formalin-induced spontaneous pain behaviors. Compared with NS and CFA+MOR rats, licking behaviors in CFA-treated rats were significantly enhanced in phase II (11–60 min), but not phase I (0–10 min). (C) Cumulative licking time in the formalin test. CFA: complete Freund's adjuvant; NS: normal saline; MOR: morphine. *, **, ***: *P*<0.05, *P*<0.01, and *P*<0.001, respectively, compared with the NS group. #, ##, and ###, *P*<0.05, *P*<0.01, and *P*<0.001, respectively, compared with the CFA+MOR group.

In experiment 1, we examined the effect of pain experience on later responses to formalin-induced spontaneous pain in rats. Inflammatory pain was produced by CFA, s.c. and evaluated by measuring thermal hyperalgesia in the CFA-injected paw. As shown in Figure 2A, intraplantar injection of CFA induced a significant decrease in PWL, compared to saline controls and the pre-CFA baseline (two-way ANOVA, group effect: *F* (2, 79) = 73.88, *P*<0.0001; time effect: *F* (5, 395) = 25.54, *P*<0.0001; interaction: *F* (10, 395) = 22.47, *P*<0.0001). Thermal hyperalgesia persisted from day 1 through 14 and recovered at day 28 post-inoculation. Early treatment with morphine completely prevented the development of hyperalgesia, as demonstrated by stable withdrawal thresholds in the CFA+MOR group over the 28-day observation period (Fig. 2A).

After recovery from inflammatory pain, rats received formalin, s.c. into the unaffected hind paw. Formalin injection resulted in a typical biphasic pattern of licking behavior in all three groups, as shown in Figure 2B. Importantly, licking behaviors of rats in the CFA group were significantly enhanced relative to the NS and CFA+MOR groups (two-way ANOVA, group effect: *F* (2, 21) = 28.03, *P*<0.0001; interaction: *F* (22, 231) = 1.94, *P*<0.01). Cumulative licking time showed a striking increase in phase II (11 to 60 min, 738.9±39.1 vs. 391.4±32.4 and 473.1±26.7 s, respectively, *P*<0.001) but not in phase I (0 to 10 min, 152.2±17.5 vs. 106.7±15.4 and 107.4±17.1 s, respectively, *P*>0.05) in CFA-treated rats (ANOVA followed by Student-Newman-Keuls test, see Fig. 2C). These results suggest that pre-exposure to persistent pain may increase the susceptibility to environmental injuries and magnify the negative perception of those injuries.

### Chronic pain experience enhances fear conditioning induced by noxious stimulation

In experiment 2, we examined the effect of inflammatory pain on later anticipatory responses.

#### Baseline reaction to tone and laser stimulation

**Figure 3:**
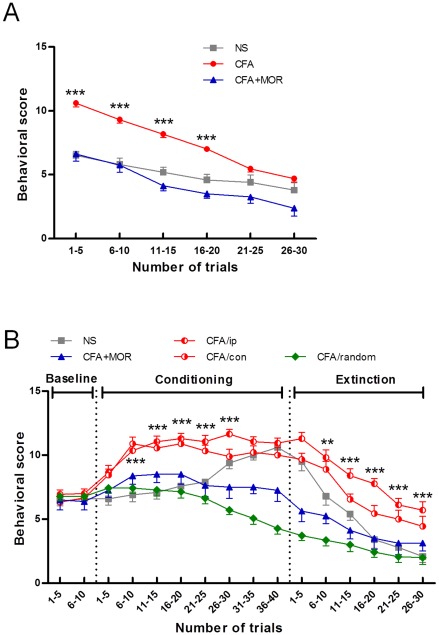
Effects of chronic pain experience on the formation test and extinction of tone-laser conditioning. (A) Orientating responses (ORs) and habituation. Relative to the other two groups, rats in the CFA group displayed more intense ORs following tone presentation, which gradually diminished in the final 10 trials. (B) Tone-induced behavioral responses during baseline, conditioning, and extinction sessions. Scores in the pairing and testing trials in the CFA/ip group were significantly higher than those in the NS, CFA+MOR, and CFA/random (unpaired CS-US) groups. There were no significant differences in behavioral scores between CFA/ip and CFA/con rats across all trials. **, ***: *P*<0.01 and *P*<0.001, respectively, CFA/ip vs. NS.

After animals recovered from prior inflammatory insults, aversive Pavlovian conditioning was conducted in which a non-aversive auditory stimulus (the CS) was followed by a noxious laser stimulus (the US). In the baseline test, the orienting response elicited by the tone was recorded, including head turning, ear pricking, rearing, and even flinching. Initially, a more intense orienting response was observed in CFA rats relative to NS or CFA+MOR rats (interaction: *F* (10, 275) = 4.30, *P*<0.001, see Fig. 3A). After repetition of the auditory stimulus, however, no difference was found among the three groups in the last 10 trials.

Laser-induced nociception was measured by a ramping procedure, in which the threshold laser intensity was determined for each individual rat. The hind paws ipsilateral (CFA/ip) and contralateral (CFA/con) to the previous CFA injection were tested separately. A one-way ANOVA revealed no significant differences in the withdrawal thresholds among NS, CFA/ip, CFA/con, CFA+MOR, and CFA/random groups (data not shown).

#### Changes in anticipatory pain behaviors after inflammatory pain experience

Responses to the tone cue alone during tone-laser pairing (i.e., conditioning) and testing (i.e., acute extinction) are shown in Figure 3B. With repeated tone-laser pairings, rats in both CFA groups and the saline group exhibited development of an anticipatory conditional orienting response. It should be noted that the anticipatory responses in the CFA/conditioning rats were established within 10 trials of pairing, while in the NS rats, they were established after 26 trials, and they were never established in the CFA/random rats (group×time interaction, *F* (60, 795) = 7.45, *P*<0.0001; group effect, *F* (4, 53) = 33.40, *P*<0.0001, time effect: *F* (15, 795) = 93.03, *P*<0.0001, see Fig. 3B). A significantly stronger and earlier anticipatory response was revealed in the CFA/ip group at trials 6–30 and in the CFA/con group at trials 6–25, in contrast with the NS group (*Student-Newman-Keuls* test, all *P*<0.01). Even in the testing (extinction) session, the CFA/ip rats maintained a higher response to tone stimulation (trials 6–30) compared to the NS group, which dropped rapidly towards zero. Interestingly, rats in the CFA+MOR group failed to acquire the conditioned response, evident as a non-significant increase in the initial 6–20 pairing trials. In addition, there were no significant differences between the CFA/ip and CFA/con rats over pairing or testing sessions.

**Figure 4:**
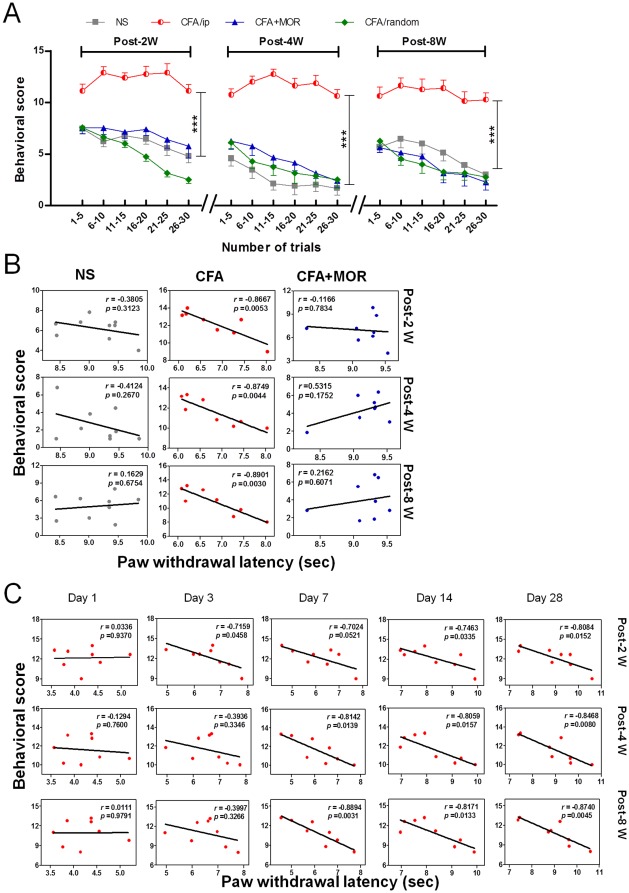
Effect of pain experience on long-term retention of conditioned responses. (A) Long-term retention of tone-induced responses in CFA rats throughout the 8-week period following conditioning. ***: *P*<0.001, compared with the NS, CFA+MOR, and CFA/random groups. (B) Correlations between mean PWLs during recovery of CFA and conditioned responses during long-term retention. Significant negative correlations were observed only in CFA-treated rats. (C) Temporal dynamics of correlations between thermal hyperalgesia and retention responses in CFA-injected rats. Significant correlations were observed from day 7–28 after CFA inflammation.

To fully address whether pain experience can cause a prolonged response to new pain-related events, we examined retention of the conditioned orienting response at 2, 4, and 8 weeks after the acute extinction session without any further exposure to the conditioning paradigm. The results are shown in Figure 4A. Remarkable tone-evoked “nocifensive” behaviors (e.g., flinching, foot elevation, licking, and even body movement) were observed in the CFA/ip group throughout the 8 weeks of retention testing. These rats displayed significantly higher behavioral scores compared to the NS, CFA+MOR, and CFA/random groups (group effect, *F* (3, 35) = 42.62, *P*<0.0001, at 2 weeks; *F* (3, 29) = 42.65, *P*<0.0001, at 4 weeks; *F* (3, 29) = 23.85, *P*<0.0001, at 8 weeks). Unlike animals in the other three groups, CFA-treated rats exhibited a high level of conditioned orienting with no decrement in responding during the course of extinction (group×time interaction, *F* (15, 175) = 7.42, *P*<0.0001, at 2 weeks; *F* (15, 145) = 3.98, *P*<0.0001, at 4 weeks; *F* (15, 145) = 2.08, *P* = 0.014, at 8 weeks), indicating maintenance of a high degree of negative emotional and/or cognitive responses due to early chronic pain experience.

#### Correlation between chronic pain experience and long-term anticipatory behavior

*Pearson* correlation coefficients were calculated to assess the relationship between thermal hyperalgesia experience and later retention of conditioned behavior (Fig. 4B and 4C). Significant negative correlations were found between average PWL during earlier inflammation and later tone-induced behavior only in CFA rats for all retention sessions (2 weeks, *r* = −0.8667, *P* = 0.0053; 4 weeks, *r* = −0.8749, *P* = 0.0044; 8 weeks, *r* = −0.8901, *P* = 0.0030, see Fig. 4B). These results suggest that more intense early pain experiences are associated with more persistent acquired pain-related responses. In contrast, this phenomenon was not observed in either the NS or CFA+MOR groups.

To determine which period of inflammatory pain can best predict later aversive conditioning, we further investigated correlations between CFA-induced thermal hyperalgesia at different time points and later retention behavior at 2 though 8 weeks (Fig. 4C). A stage-specific effect of pain history was found on long-term pain-related anticipation. Significant negative correlations were only found at the late stage of CFA-induced inflammation (day 7–28). These results further corroborated the contribution of pain history to maintenance of long-term negative emotion and cognition.

## Discussion

The present study investigated the effects of inflammatory pain history on subsequent formalin-evoked pain behaviors, as well as pain-related anticipatory responses, in adult male rats. We confirmed with a CFA inflammatory pain model that chronic pain experience in adulthood can reshape pain-related behavior in later life, which is in agreement with Hummel et al. (2008) [18]. We found that after recovery from CFA-induced hyperalgesia, paw licking behavior in response to formalin injection was elevated specifically in the second phase, which is presumed to have a greater motivational-affective component [30]. Furthermore, we obtained evidence that rats with pain experience showed enhanced acquisition and persistence of anticipatory responses to threatening stimuli, indicating that chronic pain history was associated with a subsequent higher level of vigilance against potential threats. In addition, we demonstrated that hyperalgesia in the late phase of the initial inflammatory pain correlated with sustained high vigilance. Finally, we found that the aforementioned behavioral changes resulting from pain experience were effectively prevented by morphine treatment at an early stage of chronic pain.

### Prior pain experience influences affective pain processing

One of the central findings of this study was that phase II paw-licking behavior in the formalin model was exacerbated in rats with a history of inflammatory pain, even after recovery of CFA-induced hyperalgesia. This result supports previous findings that the second phase of the formalin test occurred earlier in neonatal CFA-treated rats [15]. It has been suggested that biphasic behaviors in the formalin test depend on direct chemical activation of nociceptors in the early phase, but on ongoing nociceptor activity coupled with central sensitization in the late phase [31], [32]. Thus, pre-exposure to inflammatory pain may have facilitated subsequent central sensitization rather than location-specific nociceptive afferents.

Since the second phase of formalin pain has a relatively longer duration, it is presumed to have a greater motivational-affective component than momentary pain [30]. For example, lidocaine injections into limbic areas, such as the lateral hypothalamus [33], reticular nuclei [34], and cingulum bundle [32], [34], have been shown to produce analgesia in the formalin test but not in the foot-flick test. Vaccarino and Melzack [34] maintained that the response in the formalin test may involve integration of motivational and affective behaviors to protect the injured area. Evidence from electrophysiological studies suggests that formalin injection induced prolonged neuronal activity in the thalamocortical medial pain pathway [35]. Thus, prior inflammatory pain may selectively intensify the affective-motivational rather than the nociceptive dimension of subsequent pain.

In further support of this argument, no differences were observed between rats with and without pain history in withdrawal thresholds measured by noxious thermal stimuli one month after CFA/NS injection, whereas the tone cue elicited nocifensive responses were significantly enhanced by pre-exposure to inflammatory pain. Enhanced conditioning in CFA-treated rats may be a result of enhanced processing of the US, given that the asymptotic level of conditioning is not increased in CFA-treated rats. In other words, it is the affective dimension of the painful US that supports fear conditioning [36]. It is not possible that sustained enhanced responding to the CS was a result of long-term sensitization because rats receiving pseudo-conditioning (CFA/random group) failed to develop a conditioned response, in contrast to those receiving conditioning training, although both groups experienced chronic inflammatory pain. The difference in extinction between NS and CFA groups immediately following conditioning may not be surprising because the CFA group exhibited asymptotic conditioning for a longer period of time than the NS group. It has been suggested that learned behavior (i.e., tone-induced conditioned avoidance) in rats reflects the affective component of pain [37]. Clinical observations have demonstrated that in comparison with the pain perception per se, patients' expectancies about potential pain are more likely to be affected by previous experience [4], [38]. Using healthy human subjects, Bayer et al. [10] showed that prior exposure to ice water remarkably influenced reactivity to external suggestions of pain but did not increase the frequency of pain reports. Therefore, the present findings suggest that a history of chronic pain experience may change the cognitive aspect of pain in parallel with enhancing the negative affect. This combination may instill a strong anticipation of negative consequences. It is possible that enhanced cognitive expectation and affective reactivity not only generate more pain-related problems, but also give rise to affective disorders such as anxiety and depression, which are often found to be comorbidity with chronic pain [39], [40].

### Late-stage hyperalgesia in inflammatory pain predicts future pain vulnerability

Hypervigilance to aversive stimuli is a hallmark of functional pain disorders [41]. Consistent with previous findings [18], the current study revealed a persistent hypervigilance represented by an anticipatory response that lasted for at least 8 weeks without any additional training. A generalized hypervigilance to potentially aversive stimuli could explain heightened rates of somatic symptoms in pain patients [42]. Therefore, previous pain history, in combination with current painful accidents, may lead to prolonged pain and disability.

In the current study, we discovered that pre- and early-stage treatment with morphine fully abolished the effect of CFA-induced pain on later pain vigilance. This supports the clinical applications of preemptive analgesia [43], [44], [45], as well as recent findings that pre-emptive morphine analgesia can attenuate long-term consequences of neonatal inflammation in rats [46].

The current studies revealed a significant correlation between late-stage hyperalgesia (days 7–28 following CFA injection) and long-term retention of the conditioned response. Supporting evidence from clinical studies indicates that the chronicity of pain involves more psychological components than acute pain [47], [48] and is meaningfully related to future disability or distress [49], [50], [51]. In contrast, we failed to find any link between early-stage hyperalgesia (days 1–3) and later retention behavior. Similar results were reported in a very recent work showing that pain sensitivity in ‘tail-flick’ and ‘flinch-jump’ tests was unrelated to the conditioned fear response in normal rats [19]. Therefore, we propose that the degree of late-stage hyperalgesia during chronic pain may be a good predictor of long-term pain-related hypervigilance.

### Possible neurophysiological and psychological mechanisms

One mechanism that may underlie the phenomenon whereby a history of chronic pain increases the risk of future painful diseases is long-term plastic changes in the central neural circuits that process pain [3], [52], [53]. It is believed that development of chronic pain is a progressive process from initial changes in presynaptic release and postsynaptic receptor modifications to a final reorganization of cortical networks [54]. Our current study revealed that early treatment with morphine fully prevented hypersusceptibility to subsequent formalin insult and hypervigilance to threat-indicating cues. This was consistent with previous reports that early pain management plays a crucial role in preventing long-lasting alterations of central pain processing [55], [56], [57]. However, it is still unclear why an acute analgesic dose of morphine affects the acquisition of fear conditioning as long as one month after administration. By employing classical conditioning of the rabbit's nictitating membrane response, Schindler and colleagues found that morphine significantly retarded (1 and 5 mg/kg) or completely blocked (10 mg/kg) acquisition of conditioned responses [58], [59]. Most importantly, retarded or blocked acquisition of conditioned responses could still be detected when rabbits were tested 5 days after cessation of morphine drug injections. This is consistent with our current results. Additionally, in a recent study, Nugent et al. found that even a single dose of morphine was potent enough to impede long-term potentiation of GABA_A_-mediated synaptic transmission, even after 24 h when drug effects are no longer thought to be present [60]. Additional studies are warranted to further investigate this question.

Cognitive processing may also underlie this phenomenon. The association between previous pain experience and long-term hyper-reactivity may reflect implicit memory, in which previous experiences alter responses without conscious awareness [61]. It is presumed that chronic pain states lead to not only the development of explicit somatosensory pain memories that manifest in plastic alterations in brain areas related to pain, but also that more widespread implicit memories are created in response to psychological processes, such as operant or classical conditioning [11]. This implicit memory trace, which takes the form of abstract representations [62] and is invulnerable to disruption [63], [64], [65], may enhance pain sensitization [66] and even lead to pain perception in the absence of peripheral stimulation [67], [68]. In animal studies, conditioned emotion induced by a single cue is usually used to represent implicit memory [69], [70]. In the current study, conditioned responses observed in pain-experienced rats were invulnerable to extinction, which is characteristic of implicit memory [71]. Thus, experience of chronic pain may alter the implicit content of pain concepts, hence changing sensitivity to pain-related learning and response patterns.

In conclusion, inflammatory pain history exacerbated subsequent spontaneous pain and exerted facilitatory effects on long-term negative affective responses to pain-related cues in adult rats. Furthermore, the extent of hyperalgesia at the late stage of inflammatory pain was able to predict future emotional dysregulation. Our findings highlighted the importance of early pain control in preventing long-term effects on pain perception and expectation. A limitation of this study was that it did not examine sex differences, as the results are based only on male rats. Further studies should extend these findings by including females.
